# A multilevel meta-analysis of the effects of exercise interventions on inhibitory control in children with ADHD

**DOI:** 10.3389/fpsyt.2026.1742882

**Published:** 2026-02-17

**Authors:** Haozhi Wang, Shanshan Wang, Gong Cheng

**Affiliations:** 1 School of Physical Education, Beijing Normal University, Beijing, China; 2 School of Journalism and Communication, Northwest Minzu University, Lanzhou, China; 3 School of Physical Education, Northwest Normal University, Lanzhou, China

**Keywords:** attention-deficit/hyperactivity disorder, children, exercise intervention, inhibitory control, meta-analysis, three-level model

## Abstract

**Background:**

Inhibitory control deficits are core cognitive dysfunctions in children with attention-deficit/hyperactivity disorder (ADHD). Exercise interventions, as a non-pharmacological approach, show promise for improving executive functions, yet quantitative evidence regarding their effectiveness and moderating factors remains limited.

**Methods:**

We systematically searched PubMed, Web of Science, Embase, and the Cochrane Library from inception to October 2025. Randomized controlled trials examining exercise effects on inhibitory control in children with ADHD were included. Risk of bias was assessed using the RoB 2 tool. A three-level random-effects model was employed to pool effect sizes while accounting for within-study dependencies. Meta-regression analyses examined moderating effects of gender ratio, training frequency, session duration, and intervention duration. Evidence quality was evaluated using GRADE.

**Results:**

Eleven trials (512 participants; 268 intervention, 244 control) yielded 15 effect sizes. The three-level meta-analysis revealed a medium-to-large beneficial effect of exercise on inhibitory control (SMD = 0.71, 95% CI [0.52, 0.91], p < 0.001) with negligible heterogeneity (I² = 0%). Within-group analyses showed significant improvements in intervention groups (SMD = 0.98, 95% CI [0.68, 1.28], p < 0.001), but not in controls (SMD = 0.13, 95% CI [−0.06, 0.31], p = 0.158). Meta-regression found no significant moderators (all p > 0.05). Sensitivity analyses confirmed robust results. Egger’s test indicated no publication bias (p = 0.606). GRADE assessment indicated low-quality evidence due to lack of blinding and trial preregistration.

**Conclusion:**

Exercise interventions may produce medium-to-large improvements in inhibitory control among children with ADHD. Although robust across intervention parameters, overall evidence certainty remains low due to methodological limitations. High-quality studies are needed to confirm these findings.

**Systematic Review Registration:**

https://www.crd.york.ac.uk/prospero/, identifier CRD420251178371.

## 1 Introduction

Attention-deficit/hyperactivity disorder (ADHD) is a neurodevelopmental disorder primarily characterized by age-inappropriate patterns of inattention, hyperactivity, and impulsivity. According to the diagnostic criteria outlined in the Diagnostic and Statistical Manual of Mental Disorders, Fifth Edition (DSM-5), core symptoms of ADHD must emerge before 12 years of age, persist for at least 6 months, and cause significant impairment in social, academic, or occupational functioning across two or more settings (1) The disorder is classified into three subtypes: predominantly inattentive presentation, predominantly hyperactive-impulsive presentation, and combined presentation. The World Health Organization’s International Classification of Diseases, Eleventh Revision (ICD-11) categorizes ADHD as a neurodevelopmental disorder, similarly emphasizing the persistence, pervasiveness, and substantial functional impact of symptoms (2). ADHD affects approximately 5-7% of school-aged children, making it one of the most common neurodevelopmental disorders in childhood (3). A core deficit in ADHD is impaired inhibitory control, defined as the diminished ability to suppress prepotent responses or filter interfering information, which directly affects children’s academic performance, social interactions, and emotional regulation.

From an epidemiological perspective, ADHD exhibits significant global distribution patterns and complex demographic variations. A large-scale systematic review encompassing 102 studies worldwide reported a pooled global prevalence of 5.29% among children and adolescents, with notable regional differences—North America demonstrating the highest rates (approximately 7.2%), while the Middle East and Africa showing relatively lower prevalence (approximately 2-3%) (3). In China, a nationwide epidemiological survey across 31 provinces revealed a prevalence of 6.26% among children and adolescents, consistent with global estimates (4). Notably, ADHD shows pronounced gender disparities, with boys being 2–3 times more likely to be affected than girls, potentially reflecting the combined influence of biological factors and diagnostic biases (5).

ADHD exerts profound and enduring impacts at both individual and societal levels, with its burden extending far beyond the disorder’s symptomatic manifestations. At the individual level, children with ADHD experience multidimensional functional impairments and developmental challenges. Academically, deficits in inhibitory control impede children’s ability to sustain attention, resist distractions, and control impulsive behaviors, directly compromising classroom learning efficiency, homework completion quality, and examination performance. Consequently, children with ADHD face significantly elevated risks of academic failure, with higher rates of grade retention and school dropout compared to their peers (5). In terms of social functioning, impulsivity and emotional dysregulation contribute to frequent conflicts during peer interactions, manifesting as aggressive behaviors, difficulty adhering to social norms, and challenges maintaining friendships, resulting in widespread peer rejection and social isolation (6). Regarding mental health, children with ADHD demonstrate exceptionally high comorbidity rates with other psychiatric disorders, with approximately 50-70% presenting with at least one additional condition, including oppositional defiant disorder, conduct disorder, anxiety disorders, and depression. These comorbidities further deteriorate functional capacity and quality of life (7). More critically, ADHD symptoms often persist into adolescence and adulthood. Longitudinal studies indicate that approximately 60-80% of childhood ADHD symptoms continue into adulthood, affecting career development, marital relationships, substance abuse risk, and criminal behavior propensity, substantially reducing lifetime socioeconomic status (8). At the societal level, the burden imposed by ADHD is equally substantial. In terms of family caregiving, raising a child with ADHD places enormous psychological stress and demands considerable time and energy from parents. Research demonstrates that parents of children with ADHD experience depression and anxiety rates exceeding four times those of parents of typically developing children, with significantly elevated risks of marital conflict and family dysfunction (9). Economically, a U.S. study estimated the annual societal economic cost of ADHD at $143–266 billion, encompassing direct medical expenses, special education expenditures, productivity losses, and crime-related costs (10). Educational systems bear the burden of providing individualized support and special education resources for students with ADHD, while public health systems confront multiple challenges including insufficient diagnostic capacity, inequitable treatment resource allocation, and difficulties in long-term follow-up management (11). Therefore, exploring effective ADHD intervention strategies, particularly non-pharmacological approaches capable of improving core cognitive deficits such as inhibitory control, holds significant practical importance and public health value for alleviating individual suffering, reducing family burden, and optimizing societal resource allocation.

Current intervention strategies for ADHD primarily encompass pharmacological treatment, behavioral therapy, and educational support, yet these conventional approaches present numerous limitations. Regarding pharmacological treatment, central nervous system stimulants such as methylphenidate and amphetamine-based medications, while effectively controlling core ADHD symptoms in the short term, are associated with significant adverse effects including appetite suppression, sleep disturbances, growth inhibition, cardiovascular risks, and potential substance dependence. These side effects substantially compromise long-term medication adherence in children (12). Additionally, approximately 30% of children with ADHD demonstrate poor response to stimulant treatment or cannot tolerate medication side effects (13). Moreover, therapeutic benefits dissipate rapidly upon medication discontinuation, lacking sustained improvement in core cognitive functions. In terms of behavioral therapy, although behavioral interventions and parent training programs demonstrate some efficacy in ameliorating behavioral problems, their impact on core cognitive deficits such as executive functions remains limited. Furthermore, these approaches incur high implementation costs, depend heavily on parent and teacher involvement, and face significant barriers to widespread dissemination in resource-constrained community and school settings (14). More critically, existing empirical research yields inconsistent findings regarding the effects of different intervention modalities on inhibitory control in children with ADHD, lacking systematic quantitative synthesis and evaluation. This evidence gap undermines clinical practice and educational policy formulation with insufficient evidence-based guidance. In recent years, exercise interventions as a non-pharmacological approach have garnered increasing attention from researchers and clinicians. Exercise interventions offer numerous distinctive advantages, including absence of pharmacological side effects, strong accessibility, low cost, ease of implementation in school and community settings, and promotion of overall physical and mental health (15) Neuroscientific research indicates that regular physical activity can enhance neural circuit functions associated with inhibitory control by augmenting prefrontal cortex blood flow, promoting neurotrophic factor release, and optimizing neurotransmitter systems such as dopamine and norepinephrine (16). Although multiple empirical studies have investigated the effects of exercise interventions on cognitive functions in children with ADHD, these studies exhibit substantial heterogeneity in intervention types (aerobic exercise, resistance training, coordination training, team sports, etc.), intervention intensity, frequency, duration, and study design quality, resulting in inconsistent conclusions that necessitate integration and quantitative evaluation through systematic meta-analytic methods.

Previous meta-analyses examining the effects of exercise interventions on children with ADHD suffer from significant methodological limitations. First, most published meta-analyses employ traditional univariate models, which assume that each study contributes only one independent effect size. However, many studies actually report multiple outcome measures (e.g., different inhibitory control tasks) or conduct repeated measurements at different time points. These effect sizes exhibit statistical dependency, and univariate models cannot appropriately handle such nested data structures, potentially leading to biased standard error estimates and inflated Type I error rates (16). Second, existing reviews typically include small sample sizes, studies with high design heterogeneity and inconsistent methodological quality, and lack systematic assessment of publication bias and study quality (17). Third, most meta-analyses fail to adequately explore potential moderating variables, such as exercise type (aerobic vs. skill-based exercise), intervention dosage (frequency, intensity, duration), and participant characteristics (age, gender, ADHD subtype, medication status), limiting their ability to address the critical clinical question of “what type of exercise, under what conditions, is most effective for which children with ADHD” (18). The present study employs multilevel meta-analysis, a more advanced statistical technique capable of simultaneously modeling between-study and within-study variance components, appropriately handling nested structures and statistical dependencies among effect sizes, thereby providing more precise effect size estimates and more reliable inferences (19) By comprehensively considering multidimensional characteristics of exercise interventions (type, intensity, frequency, total duration) as well as participants’ demographic and clinical features, this study will systematically explore the potential moderating effects of these variables on intervention outcomes, providing an empirical foundation for developing personalized exercise intervention protocols.

Based on the foregoing background, this study aims to systematically integrate existing literature evidence through multilevel meta-analysis to quantitatively evaluate the effects of exercise interventions on inhibitory control in children with ADHD. Specifically, the objectives of this study include: first, estimating the overall effect size and its 95% confidence interval for exercise interventions on inhibitory control in children with ADHD, clarifying the overall effectiveness of exercise interventions in improving this core cognitive deficit; and enhancing the methodological rigor of meta-analysis and accuracy of statistical inference by employing multilevel modeling techniques to address statistical dependencies among effect sizes. The findings of this study will provide evidence-based guidance for clinicians, educators, parents, and policymakers regarding exercise interventions as a non-pharmacological treatment option for children with ADHD.

## 2 Methods

### 2.1 Study design and registration

The protocol for this systematic review and meta-analysis was prospectively registered in the International Prospective Register of Systematic Reviews (PROSPERO; registration number: CRD420251178371) to ensure transparency and reproducibility of the research process and to minimize the risk of selective reporting. The design, conduct, and reporting of this study strictly adhered to the Preferred Reporting Items for Systematic Reviews and Meta-Analyses (PRISMA) 2020 statement.

### 2.2 Search strategy

This study employed a systematic search strategy to comprehensively search four major international academic databases: PubMed, Web of Science Core Collection, Embase, and the Cochrane Library. The search covered the period from database inception to October 2025, with no language restrictions, to maximize identification of all relevant studies. The search strategy was collaboratively developed by experienced researchers and medical library specialists, combining controlled vocabulary with free-text terms. For PubMed, standardized searches were conducted using Medical Subject Headings (MeSH), and search formulas were appropriately adapted according to the characteristics of different databases to ensure comprehensiveness and precision. The core search concepts encompassed three dimensions: population (“ADHD,” “attention deficit hyperactivity disorder,” “hyperkinetic disorder,” etc.), intervention (“exercise,” “physical activity,” “sports,” “aerobic training,” etc.), and outcomes (“inhibitory control,” “executive function,” “cognitive control,” “response inhibition,” etc.). Boolean logic operators (AND, OR) were used to combine search terms, with appropriate truncation and proximity operators employed to enhance search sensitivity. Additionally, to supplement potentially missed studies from electronic database searches, the research team manually searched reference lists of included studies and relevant systematic reviews, and conducted supplementary searches through search engines such as Google Scholar to ensure completeness of literature inclusion.

### 2.3 Inclusion and exclusion criteria

Inclusion criteria were systematically established based on the PICOS framework (Population, Intervention, Comparison, Outcome, Study design). Regarding population (P), studies were included if they enrolled children and adolescents (aged 5–18 years) with a confirmed diagnosis of ADHD according to internationally recognized diagnostic criteria (e.g., DSM-5, DSM-IV, or ICD-10), without restrictions on ADHD subtype, comorbidity status, or medication treatment status. Regarding intervention (I), studies employing any form of structured exercise intervention were included, encompassing but not limited to aerobic exercise, resistance training, ball sports, swimming, roller skating, and exercise games, with no restrictions on intervention frequency, intensity, duration, or implementation setting. Regarding comparison (C), studies utilizing usual care, no intervention, or waitlist controls were included to evaluate the net effects of exercise interventions. Regarding outcomes (O), studies reporting measurements related to inhibitory control were included, encompassing inhibitory control functions assessed using standardized neuropsychological tasks (e.g., Stroop task, Go/No-Go task, Flanker task, Simon task, Stop-Signal task) or behavioral rating scales. Regarding study design (S), randomized controlled trials (RCTs) were included.

Exclusion criteria provided further refinement and specification beyond the inclusion conditions, aiming to enhance study homogeneity and methodological quality. Specifically, studies were excluded if: (1) they involved multicomponent interventions (e.g., exercise combined with medication or dietary interventions) without the ability to isolate exercise effects; (2) they were non-original research publications such as conference abstracts, dissertations, case reports, or review articles; (3) they lacked sufficient statistical data (e.g., means, standard deviations, sample sizes) and supplementary data could not be obtained through author contact; or (4) they had ambiguously defined outcome measures or assessment methods inconsistent with the concept of inhibitory control, thereby avoiding interference with analytical results due to conceptual ambiguity or data insufficiency.

### 2.4 Literature screening and data collection

The literature screening and data extraction processes strictly adhered to systematic review methodological standards to ensure objectivity and accuracy in study selection and data collection. Literature screening employed a dual independent assessment mechanism, whereby two researchers independently conducted a three-stage screening process comprising title screening, abstract screening, and full-text evaluation. EndNote X20 reference management software was used for literature organization and duplicate removal. At each screening stage, the two assessors independently determined whether studies met the predefined inclusion and exclusion criteria. For studies with discrepancies, consensus was reached through discussion; if agreement could not be achieved, a third senior researcher provided the final adjudication to ensure reliability and consistency of literature screening. Data extraction similarly employed dual independent operation with mutual cross-checking, utilizing pre-designed and pilot-tested standardized data extraction forms to systematically extract the following information: (1) study design and basic information, including authors, publication year, country/region, and study type; (2) participant characteristics, including sample size, age, gender distribution, ADHD diagnostic criteria, ADHD subtype, comorbidity status, and medication use status; (3) specific intervention details, including exercise type, intervention frequency (sessions per week), session duration (minutes), intervention duration (weeks), intervention intensity, implementation setting, and supervision mode; (4) control group configuration and implementation details; and (5) assessment methods and outcome measures, including specific names of inhibitory control measurement instruments, testing time points, and effect indicators (means, standard deviations, sample sizes). For data presented in graphical formats, professional tools such as WebPlotDigitizer were employed to extract numerical values to ensure data precision. For studies with missing or unclear data, authors were contacted via email to obtain supplementary information; studies were excluded if authors did not respond or could not provide the required data.

### 2.5 Risk of bias evaluation

The methodological quality and risk of bias assessment of included studies employed multiple tools for systematic evaluation. This study utilized the risk of bias assessment tool recommended by the Cochrane Collaboration (20) to systematically evaluate the risk of bias in included literature. Specifically, RCTs were assessed using the Risk of Bias tool for randomized trials, version 2 (RoB 2) (21), which evaluates five bias domains: bias arising from the randomization process, bias due to deviations from intended interventions, bias due to missing outcome data, bias in measurement of the outcome, and bias in selection of the reported result. Each domain was judged as “low risk,” “some concerns,” or “high risk” based on responses to relevant signaling questions, with the overall risk of bias rating determined by synthesizing assessments across all domains. All quality assessments and risk of bias evaluations were completed by two independent assessors, with disagreements resolved through discussion or third-party adjudication.

### 2.6 Data conversion and effect size calculation

This study employed standardized mean difference (SMD) as the effect size metric to integrate effect estimates from different measurement instruments. For within-group pre-post comparisons, the mean difference between pre- and post-intervention was first calculated, from which the standard deviation of change was derived. Since extremely few included studies reported correlation coefficients between pre- and post-measurements, this study adopted the conservative recommendation from the Cochrane Handbook for Systematic Reviews of Interventions by setting the correlation coefficient r = 0.5 in the primary analysis, with sensitivity analyses conducted using r values of 0.6, 0.7, and 0.9 to examine result robustness.

For between-group comparisons, pooled standard deviation (SD_pooled) was used to synthesize variability across both groups. When studies reported only standard errors (SE) rather than standard deviations, conversions were performed based on the relationship between standard error and sample size. Considering that some studies had small sample sizes, Hedges’ g—a bias-corrected effect size proposed by Hedges and Olkin—was employed to reduce small-sample bias by applying a correction factor to Cohen’s d, thereby yielding more robust effect estimates. This effect size metric was uniformly applied to both within-group and between-group comparisons.

To ensure consistency in effect direction and accurately reflect functional improvement, this study standardized the effect directions of different measurement instruments following recommendations from the Cochrane Handbook: for indicators where lower scores represent better function (e.g., reaction time, error rate), effect sizes were multiplied by negative one; for indicators where higher scores represent better function (e.g., accuracy rate, positive scores on behavioral rating scales), the original direction was retained. After standardization, all positive effects represented functional improvement.

Effect size interpretation followed Cohen’s standards: values below 0.2 indicate trivial effects, 0.2 to 0.5 indicate small effects, 0.5 to 0.8 indicate medium effects, and 0.8 or above indicate large effects. Between-study heterogeneity was assessed using the I² statistic and Cochran’s Q test, with I² values of 25%, 50%, and 75% representing low, moderate, and high heterogeneity, respectively. Significant heterogeneity was indicated when the Q test p-value was less than 0.1. To further reflect the potential range of variation in results from future similar studies, this study also calculated prediction intervals (PI) to provide more practically meaningful references for clinical practice and subsequent research.

### 2.7 Statistical analysis methods

#### 2.7.1 Three level meta-analysis

Considering that multiple effect sizes reported within the same study (e.g., different measurement time points or different inhibitory control tasks) are often correlated, directly incorporating these non-independent effect sizes into traditional two-level meta-analysis may violate the assumption of independence among effect sizes across studies. This can lead to underestimated standard errors, overly narrow confidence intervals, excessively liberal statistical tests, and artificially inflated I² statistics, ultimately resulting in overestimated effect sizes (22). Conversely, if a conservative strategy is adopted whereby only one effect size per study is included (e.g., selecting a single time point or single task, or calculating the average of multiple effect sizes), although this satisfies the independence assumption, such an approach is overly conservative. It not only discards substantial valuable information and reduces statistical power but also fails to authentically reflect the complete distribution of intervention effects (19). Therefore, this study employed the three-level meta-analysis method proposed by Assink and colleagues (23), which fully accounts for the nested hierarchical structure of effect sizes (effect sizes nested within studies). This approach appropriately controls for within-study effect size dependencies while retaining all available information, thereby preserving multiple effect sizes within each study, enhancing statistical power, and more authentically reflecting the actual distribution of intervention effect sizes (24). This method treats multiple measurements or comparisons within the same study as the “within-study level” (Level 2), variation between different studies as the “between-study level” (Level 3), and sampling variance as the fundamental “effect size level” (Level 1), thereby decomposing the total observed variance in effect sizes into three components: sampling variance, within-study variance, and between-study variance, to control for within-study (or within-group) correlations (25).

This study implemented analysis based on the three-level modeling framework proposed by Assink and colleagues, utilizing open-source R code provided by Xu (26). All model parameters were estimated using restricted maximum likelihood (REML), with statistical significance and confidence intervals for model parameters calculated based on the t-distribution. All three-level meta-analyses were conducted using the metafor package in R (version 4.3.0). This approach not only preserves all available effect sizes reported in each study but also more comprehensively and accurately reflects the true distribution and uncertainty of intervention effects, providing stronger evidentiary support for result interpretation and intervention strategy optimization.

#### 2.7.2 Subgroup analysis and meta-regression

To explore the influence of potential moderating variables on intervention effects and explain between-study heterogeneity, this study conducted systematic subgroup analyses and meta-regression analyses. Considering that the relationship between exercise intervention effects and moderating variables may exhibit nonlinear characteristics (e.g., inverted U-shaped dose-response relationships), the linear regression assumption of unlimited growth or decline trends may not suit the actual circumstances of exercise intervention research. Therefore, this study employed comparisons between linear and various nonlinear meta-regression methods (27), including simple linear regression, quadratic and cubic polynomial regression, and restricted cubic spline (RCS) regression. The optimal model was ultimately selected based on comprehensive consideration of goodness-of-fit indicators (e.g., R², adjusted R², Akaike Information Criterion [AIC], Bayesian Information Criterion [BIC]) and practical significance (28). Regression analysis enables prediction of the influence of different variables on the dependent variable (29). All regression models were executed using the metafor package in R and visualized using the ggplot2 package.

### 2.8 Publication bias assessment

Publication bias represents a critical methodological issue in systematic reviews, referring to the phenomenon whereby the nature of study results (e.g., statistical significance, effect direction, effect size magnitude) influences publication probability, causing published literature to inadequately represent all completed studies. This can lead to biased and inaccurate meta-analysis results. This study employed multiple methods to assess publication bias risk, including visual inspection of funnel plots (30) and Egger’s regression test (31). Funnel plots display effect sizes on the horizontal axis and standard errors (or other indicators of precision) on the vertical axis. In the absence of publication bias, studies should be symmetrically distributed on both sides of the pooled effect size, forming an inverted funnel shape. Egger’s regression test quantitatively assesses publication bias by testing funnel plot asymmetry, with p > 0.05 suggesting no significant publication bias risk (32). Considering that this study employed three-level meta-analysis, wherein individual studies typically contain multiple effect sizes that are influenced by intervention parameters and exhibit mutual dependencies, and between-study effect sizes themselves differ, this study conducted publication bias assessment at two hierarchical levels: at the within-study level (Level 2) to assess publication bias across all effect sizes, and at the between-study level (Level 3) using the mean effect size of each study to assess study-level publication bias, thereby comprehensively evaluating potential selective publication issues at different levels.

### 2.9 Sensitivity analysis

To evaluate the robustness and reliability of meta-analysis results, this study conducted systematic sensitivity analyses to identify studies or effect sizes exerting undue influence on the pooled effect size and to examine the sensitivity of results to the inclusion or exclusion of individual studies. Sensitivity analyses employed multiple statistical diagnostic methods, including hat values (leverage) (33), Cook’s distance, and studentized residuals (34). These metrics were used to identify studies with extreme positions in predictor variable space (high leverage points), studies with excessive influence on model fit (high influence values), and studies with large deviations between estimated effect sizes and model-predicted values (outliers or anomalous values), respectively. Considering the hierarchical structure of three-level meta-analysis, this study conducted diagnostics separately at the within-study level (Level 2, effect size level) and the between-study level (Level 3, study level) to comprehensively identify effect sizes or studies that might affect result stability. Additionally, this study employed leave-one-out analysis, sequentially excluding each effect size or each study at both the within-study level and between-study level, recalculating the pooled effect size from the remaining data. By comparing the range of effect size variation and statistical significance across different iterative analyses, the influence of each effect size or each study on overall results was examined, thereby determining whether meta-analysis results were overly dependent on one or several studies and ensuring the robustness and generalizability of conclusions.

### 2.10 Evidence grading

To enhance transparency and reliability of result interpretation and guide clinical decision-making and future research directions, this study systematically assessed the overall quality of evidence from the meta-analysis by integrating quality ratings of all included studies. Evidence quality assessment employed the Grading of Recommendations Assessment, Development and Evaluation (GRADE) approach (35), an internationally recognized evidence grading system widely used in systematic reviews, clinical practice guidelines, and health technology assessments. GRADE classifies evidence quality into four levels—high, moderate, low, and very low—reflecting our confidence in the effect estimates. The GRADE framework encompasses five factors that may downgrade evidence quality (risk of bias, inconsistency of results, indirectness of evidence, imprecision of results, and publication bias) and three factors that may upgrade evidence quality (large effect size, presence of dose-response relationship, and all plausible confounding factors would reduce the observed effect). For RCTs, GRADE assessment begins with high-quality evidence, which is then adjusted according to each downgrading and upgrading factor to determine the final evidence quality level. For risk of bias, the aforementioned RoB 2 assessments were comprehensively considered. For inconsistency of results, evaluation primarily relied on the I² statistic, Q test p-value, and consistency in the direction and magnitude of effect estimates. For indirectness of evidence, the alignment between the PICO (Population, Intervention, Comparison, Outcome) of included studies and the research question was assessed. For imprecision of results, whether sample sizes achieved the optimal information size (OIS) and the width of confidence intervals were evaluated. For publication bias, results from funnel plots and Egger’s test were synthesized. Ultimately, this study presented GRADE assessment results in the form of a Summary of Findings table, providing clinicians, patients, and policymakers with clear and transparent information regarding evidence quality.

## 3 Results

### 3.1 Search results

**Figure 1:**
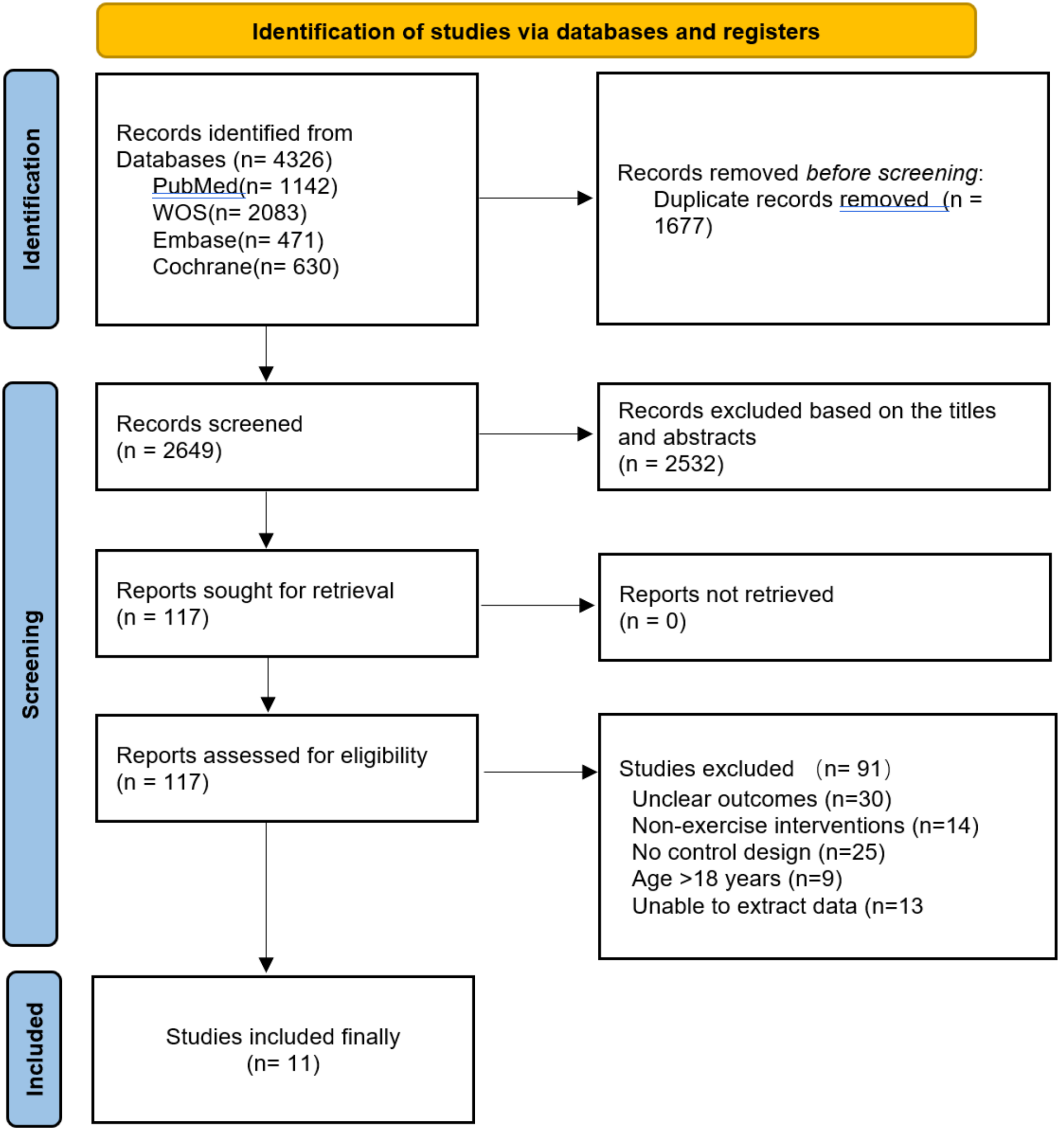
Flowchart of literature screening.

In this study, we conducted systematic searches across four major databases: PubMed (n = 1,142), Web of Science (n = 2,083), Embase (n = 471), and Cochrane Library (n = 630), yielding a total of 4,326 articles. After initial deduplication, 1,677 duplicate articles were removed, leaving 2,649 articles for title and abstract screening. Following preliminary screening, 2,532 articles irrelevant to the topic or lacking sufficient information were excluded, retaining 117 articles for full-text evaluation. During the full-text screening phase, 30 studies with unclear outcome measures, 14 non-exercise intervention studies, 25 studies without control designs, 9 studies with participants exceeding 18 years of age, and 13 studies from which effect size data could not be extracted were further excluded. Ultimately, 11 studies meeting the inclusion criteria were included in the meta-analysis. See **Figure 1** for details.

### 3.2 Characteristics of included studies

This meta-analysis included 11 RCTs comprising a total of 512 children and adolescents with ADHD (268 in intervention groups, 244 in control groups). Sample sizes of included studies ranged from 19 to 51 participants, with a median sample size of 30. Publication years spanned from 2003 to 2025, with 2 studies published in 2021, reflecting recent research activity in this field. The geographic distribution of included studies was relatively broad, encompassing multiple countries and regions: 3 studies from Iran (36 two studies by 37), 2 studies from Switzerland (38, 39), 2 studies from Taiwan (40, 41), and 1 study each from the United States, Canada, Tunisia, and South Africa. This geographic diversity enhances the external validity and generalizability of study findings.

Participant ages in included studies ranged from 5 to 14 years, primarily concentrated in school-aged children and early adolescents. All studies employed internationally recognized diagnostic criteria for ADHD, including DSM-5 (4 studies), DSM-IV (4 studies), ICD-10 (2 studies), and one study each using DSM-IV-TR and DSM-III-R, ensuring standardization and reliability of participant diagnoses. Regarding gender distribution, the proportion of male participants in included studies ranged from 48.3% to 100%, with an average of approximately 77%, consistent with the gender distribution characteristics of ADHD in childhood (higher prevalence in males than females). Concerning medication status, participants in 7 studies (63.6%) continued ADHD medication treatment (e.g., methylphenidate) during the intervention period, while 4 studies (36.4%) required participants to discontinue medication during the intervention or recruited unmedicated participants. This diversity in medication status facilitates evaluation of exercise intervention effects across different treatment contexts.

Exercise intervention types exhibited considerable diversity. Intervention frequency ranged from 1 to 5 sessions per week, with 2 to 3 sessions per week being most common (7 studies). Single session durations ranged from 10 to 90 minutes, with a median of 60 minutes. Total intervention duration ranged from single acute interventions (1 week) to 24 weeks, with a median of 10 weeks. This broad distribution of intervention parameters provides a foundation for exploring dose-response relationships. See **Appendix** for details.

### 3.3 Quality assessment of included studies

**Figure 2:**
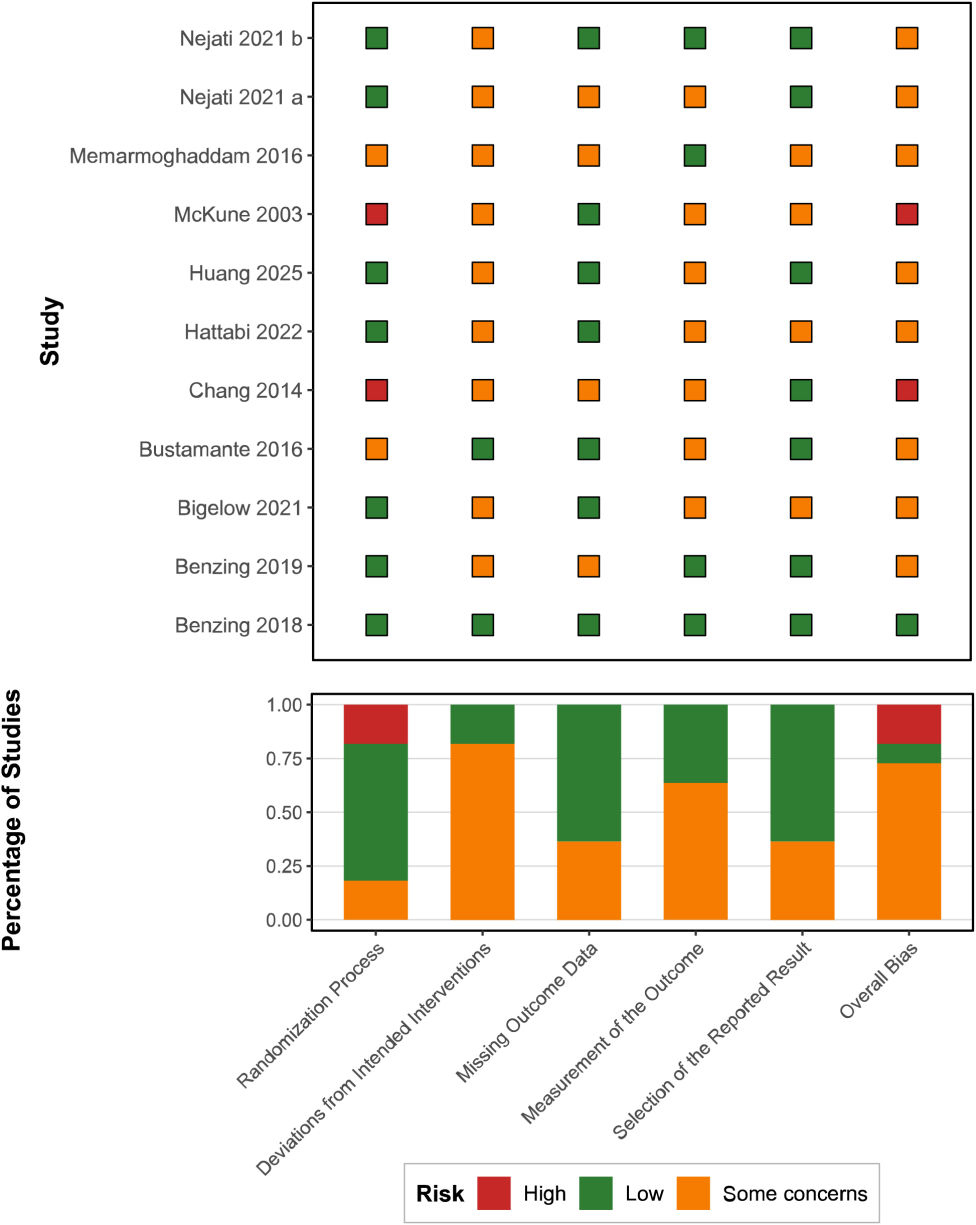
Risk of bias assessment for the included studies based on the RoB 2 tool.

Among the 11 included studies, only 1 study (38) was rated as having overall low risk of bias (9.1%); 9 studies (81.8%) raised some concerns; and 2 studies (18.2%) were assessed as high risk of bias, specifically McKune et al. (42) and Chang et al. (40). Across the five assessment domains of the RoB 2 tool, the randomization process domain demonstrated the best performance, with 9 studies (81.8%) rated as low risk. These studies reported clear randomization methods and demonstrated good baseline balance. In contrast, the domains of deviations from intended interventions and measurement of the outcome represented the primary methodological concerns, with 81.8% and 72.7% of studies raising some concerns, respectively. In the deviations from intended interventions domain, the main issue was that the nature of exercise interventions precluded blinding of participants and personnel, potentially leading to expectation effects and performance bias. In the measurement of outcome domain, the primary concern was unclear or absent outcome assessor blinding, with some studies employing subjective rating scales potentially influenced by assessor judgment. The missing outcome data domain performed well, with 90.9% of studies rated as low risk. In the selective reporting domain, 5 studies were rated as low risk, while 6 studies raised some concerns, primarily due to absence of trial pre-registration and unpublished study protocols. Notably, the control group in McKune et al. (42) comprised children unable to participate in after-school activities, representing convenience sampling rather than true random allocation. Chang et al. (40) lacked genuine randomization in the grouping process and failed to adequately control for potential confounding factors. Overall, most included studies demonstrated moderate methodological quality, with deviations from intended interventions and measurement of the outcome representing the primary methodological limitations, largely attributable to inherent difficulties in implementing blinding in exercise intervention research. See **Figure 2** for details.

### 3.4 Analysis results

Three-level meta-analysis results demonstrated that exercise interventions significantly improved inhibitory control in children with ADHD. Between-group analysis indicated that the exercise intervention group exhibited significantly better inhibitory control performance compared to the control group (SMD = 0.71, 95% CI [0.52, 0.91], p < 0.001). This analysis included 11 studies comprising 15 independent effect sizes (some studies reported results from multiple measurement time points or multiple inhibitory control tasks), with a total of 512 participants (268 in intervention groups, 244 in control groups).

Heterogeneity tests revealed extremely low between-study heterogeneity. Cochran’s Q test yielded Q(14) = 16.31, p = 0.295, failing to reach statistical significance, indicating that between-study effect size variation did not exceed the expected range of sampling error. The I² statistic was 0%, further confirming virtually no true heterogeneity between studies, with highly consistent effect estimates across all studies.

**Figure 3:**
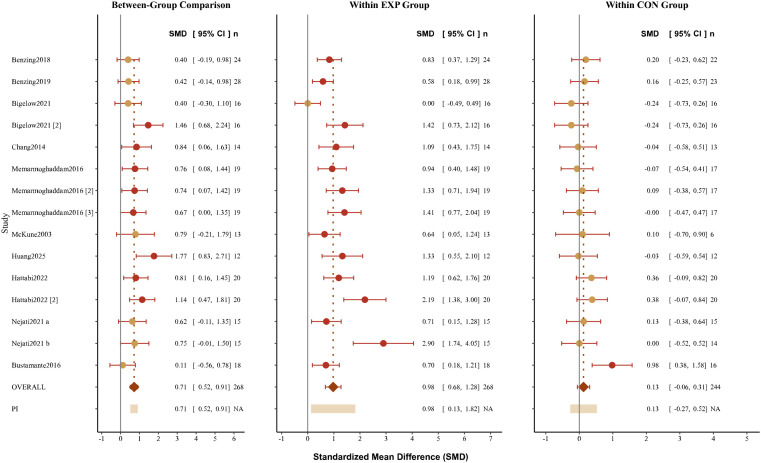
Forest plots of the standardized mean differences (SMDs) for inhibitory control outcomes.

The forest plot (**Figure 3**) visually displays the effect sizes and their 95% CIs for each study. As shown in the figure, point estimates for the 15 effect sizes ranged from 0.3 to 1.2, with all effect estimates favoring exercise interventions (positioned to the right of the line of no effect). Only 3 effect sizes had CI lower limits slightly crossing the line of no effect (SMD = 0), but their upper limits were clearly positive. The pooled effect size is represented by a diamond, centered at SMD = 0.71, with the left and right edges representing the lower and upper bounds of the 95% CI, respectively. The diamond is entirely positioned to the right of the line of no effect with a clear distance from it.

Within-group analyses further elucidated the mechanisms underlying exercise intervention effects. Pre-post within-group analysis of the experimental groups revealed significant improvement following intervention compared to baseline levels (SMD = 0.98, 95% CI [0.68, 1.28], p < 0.001). This effect size approached the threshold for a large effect (Cohen’s d ≈ 1.0), indicating that children in intervention groups experienced substantial enhancement in inhibitory control. Notably, heterogeneity in the experimental group within-group analysis was relatively high (I² = 60.62%), suggesting some variation in the magnitude of improvement across different studies. This may relate to factors such as intervention type, intervention intensity, intervention duration, and baseline severity. In contrast, within-group analysis of control groups showed no significant changes over the same time period (SMD = 0.13, 95% CI [-0.06, 0.31], p = 0.158), with the CI crossing the line of no effect, indicating that inhibitory control abilities in children in control groups did not undergo meaningful changes during the observation period. Heterogeneity in the control group within-group analysis was relatively low (I² = 29.66%). This comparative result clearly demonstrates that the observed between-group differences primarily stemmed from substantial improvements in exercise intervention groups rather than functional deterioration in control groups or significant test-retest practice effects. Integrating the findings from both between-group and within-group analyses, exercise interventions demonstrate significant, stable, and clinically valuable effects in improving inhibitory control abilities in children with ADHD.

### 3.5 Subgroup analysis

### 3.6 Meta-regression analysis

To explore the influence of potential moderating variables on exercise intervention effects, this study conducted systematic meta-regression analyses incorporating four continuous moderating variables: male percentage, sessions per week, minutes per session, and duration in weeks. These variables represented intervention participant characteristics and intervention dose parameters that might influence the effectiveness of exercise interventions in improving inhibitory control abilities in children with ADHD.

Linear meta-regression analyses revealed that none of the tested moderating variables achieved statistical significance. Specifically, male percentage showed no significant effect on effect size (β = 0.006, SE = 0.006, 95% CI [-0.007, 0.019], p = 0.368), indicating that intervention effects remained stable across samples with different gender ratios. The regression coefficient for sessions per week was negative but non-significant (β = -0.084, SE = 0.082, 95% CI [-0.245, 0.078], p = 0.311), suggesting that increasing training frequency neither significantly enhanced nor diminished intervention effects. The regression coefficient for minutes per session was positive but similarly non-significant (β = 0.004, SE = 0.003, 95% CI [-0.002, 0.009], p = 0.219), indicating limited influence of single session duration variations on effect size. The regression coefficient for duration in weeks approached zero and was non-significant (β = 0.007, SE = 0.014, 95% CI [-0.020, 0.033], p = 0.630), indicating no apparent effect of total intervention duration within the examined range (1–24 weeks). Residual heterogeneity (τ²) for all linear regression models approached zero (0.002-0.019), and QE statistics were all non-significant (p-value range: 0.246-0.321), further supporting the absence of moderating effects.

Considering that linear models might fail to capture complex dose-response relationships, this study further conducted nonlinear relationship testing. Quadratic and cubic polynomial regression analyses revealed that all higher-order terms (quadratic and cubic terms) for all moderating variables were non-significant (all p > 0.05), indicating no U-shaped, inverted U-shaped, or more complex curvilinear relationships between effect sizes and moderating variables. Restricted cubic spline (RCS) regression analyses similarly detected no significant nonlinear patterns, with spline terms in both 2-knot and 3-knot RCS models being non-significant. Locally weighted regression (Loess) analyses yielded low R² values (0.084-0.513), with adjusted R² values even becoming negative (-0.352 to 0.275), indicating that nonlinear fitting did not substantially improve model explanatory power. These results consistently demonstrate no apparent nonlinear relationships between exercise intervention effects and the examined moderating variables.

Interaction effect analyses further examined the joint effects among moderating variables. This study tested bidirectional interaction effects for six pairs of moderating variables, including the interaction between sessions per week and duration in weeks (β = -0.037, p = 0.091), male percentage and minutes per session (β = 0.001, p = 0.207), minutes per session and duration in weeks (β = 0.000, p = 0.462), male percentage and sessions per week (β = 0.006, p = 0.536), sessions per week and minutes per session (β = -0.001, p = 0.757), and male percentage and duration in weeks (β = 0.000, p = 0.969). All interaction terms failed to achieve statistical significance, with the interaction between sessions per week and duration in weeks approaching the significance threshold (p = 0.091) but remaining insufficient to support conclusions of substantial interaction effects.

Synthesizing results from linear regression, nonlinear testing, and interaction effect analyses, meta-regression analyses identified no significant moderating variables. From a methodological perspective, the extremely low between-study heterogeneity (I² = 0%) itself predicted difficulty in moderating variables explaining effect size variation, as virtually no true variation existed to explain. From a practical perspective, this finding suggests that the beneficial effects of exercise interventions on inhibitory control abilities in children with ADHD demonstrate high robustness and broad applicability, unaffected by factors such as gender composition, training frequency, session duration, or total duration. In other words, within the parameter ranges covered by this study (1–5 sessions per week, 20–60 minutes per session, 1–24 weeks duration), various intervention protocols can produce similar medium-to-large effects. This provides flexibility and options for clinical practice and future research, with intervention programs under different resource conditions and implementation settings potentially achieving favorable outcomes.

### 3.7 Sensitivity analysis

To evaluate the robustness and reliability of meta-analysis results, this study conducted systematic sensitivity analyses, including leave-one-out analysis and multiple statistical diagnostic methods to detect potential outliers and high-influence studies. These analyses aimed to examine the influence of individual studies on the pooled effect size and ensure that meta-analysis results were not overly dependent on one or several studies.

**Figure 4:**
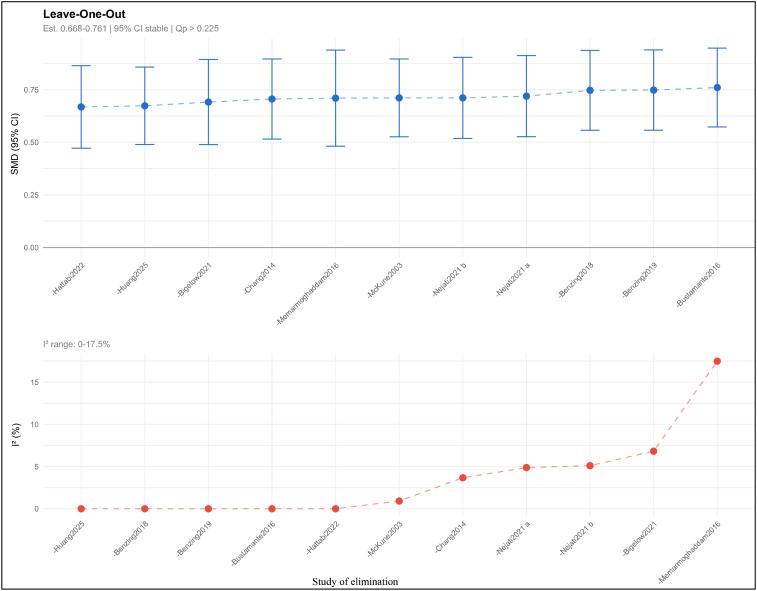
Leave-one-out sensitivity analysis for the meta-analysis of exercise interventions on inhibitory control in children with ADHD.

Leave-one-out sensitivity analysis demonstrated high stability of meta-analysis results. This method sequentially excluded each study and recalculated the pooled effect size from remaining studies, conducting 11 iterative analyses. Results indicated that after excluding any single study, the pooled effect size ranged from 0.668 to 0.760 (SMD), with a variation magnitude of only 0.092, representing minimal deviation from the original pooled effect size (SMD = 0.714). Effect sizes from all 11 iterative analyses maintained statistical significance (all p < 0.001), with 95% CIs not crossing the line of no effect. Specifically, exclusion of Huang et al. (41) yielded the smallest effect size (SMD = 0.674, 95% CI [0.489, 0.858]), while exclusion of Bustamante et al. (43) produced the largest effect size (SMD = 0.761, 95% CI [0.573, 0.948]). Notably, in most iterations, between-study heterogeneity remained at extremely low levels (I² < 5%). Only upon exclusion of Memarmoghaddam et al. (36) did I² increase slightly to 17.5%, but this still fell within the low heterogeneity category. These results consistently demonstrate that regardless of which study was excluded, the primary conclusion of the meta-analysis—that exercise interventions produce medium-to-large improvements in inhibitory control abilities in children with ADHD—remained unchanged. See **Figure 4** for details.

Cook’s distance analysis was employed to identify studies exerting excessive influence on model fit. Cook’s distance comprehensively considers both study residual magnitude and leverage, with larger values indicating greater study influence on pooled results. This study set the Cook’s distance threshold at 0.308 (based on the formula 4/(k-p-1), where k represents the number of studies and p represents the number of covariates). Analysis results revealed that all studies had Cook’s distances below this threshold, with the maximum value being 0.257 (43), followed by 0.212 (44) and 0.190 (41). Although these three studies exhibited relatively higher Cook’s distances, none exceeded the established critical value, indicating that no study exerted inappropriate excessive influence on meta-analysis results.

Standardized residual analysis was used to identify studies with large deviations between estimated effect sizes and model-predicted values. Absolute standardized residual values exceeding 1.96 are typically considered thresholds warranting attention. In this study, only one study (41) had a standardized residual slightly exceeding this threshold (standardized residual = 2.47), indicating that this study reported a relatively large effect size deviating from the model-predicted value by approximately 2.5 standard errors. However, considering that encountering one standardized residual exceeding 1.96 among 15 effect sizes is statistically expected (approximately 5% false positive rate), and that the study’s standardized residual did not reach the stringent outlier criterion of 3.0, it cannot be considered a true outlier. Furthermore, the aforementioned leave-one-out analysis demonstrated that even with exclusion of Huang et al. (41), the pooled effect size remained significant and stable, further supporting the judgment that while this study appeared somewhat anomalous, it did not affect overall conclusions.

**Figure 5:**
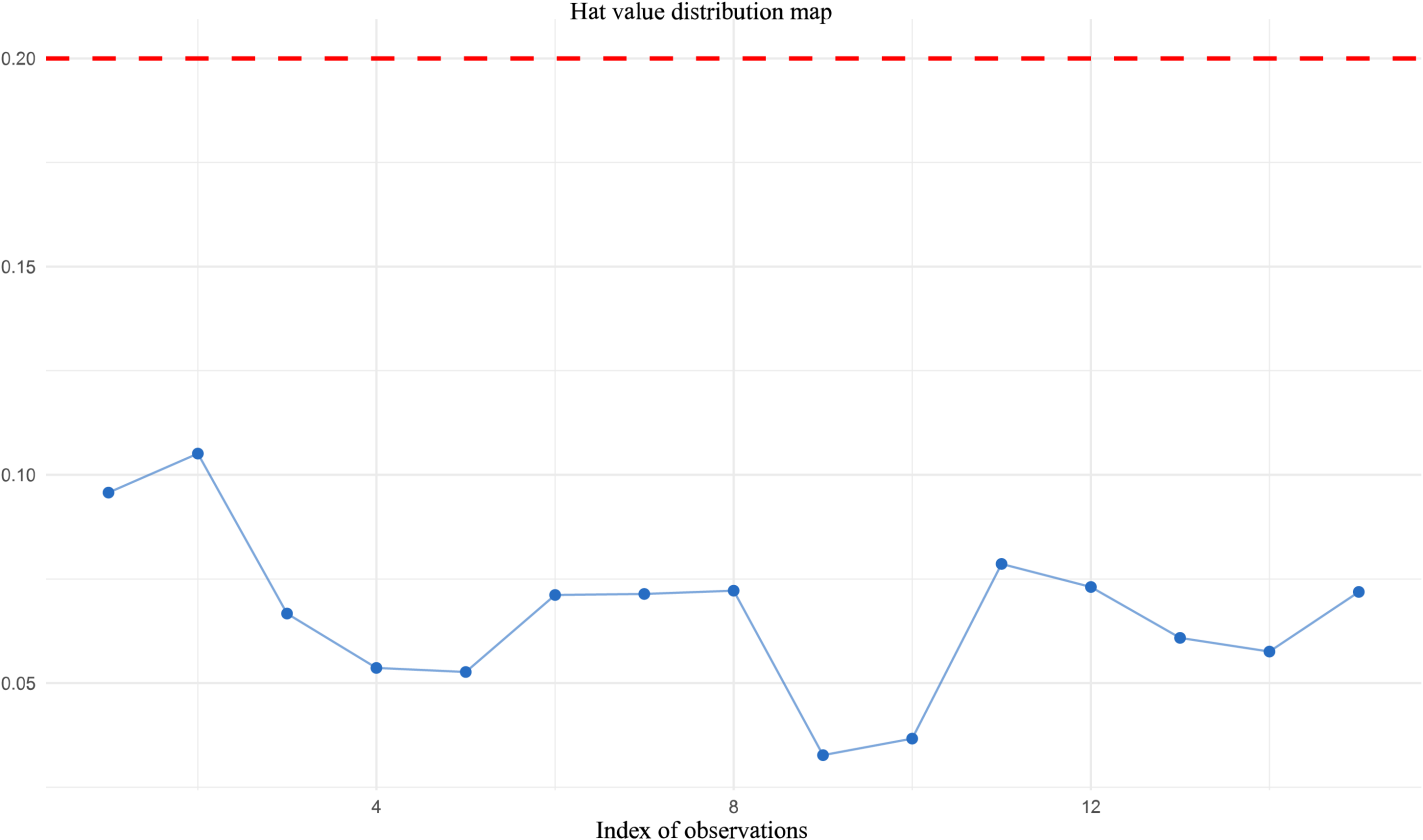
Hat value distribution map for influence diagnostics in the meta-analysis.

Hat value (leverage) analysis was employed to identify studies occupying extreme positions in moderator variable space that might exert substantial influence on regression analyses. This study set the hat value threshold at 0.2 (commonly using 2p/k or 3p/k standards). Results showed that all studies had hat values below this threshold, with the maximum hat value being 0.105 and mean hat value being 0.067, indicating that no study occupied an extreme position in moderator variable space. All studies exerted leverage on regression analyses within reasonable ranges. See **Figure 5** for details.

### 3.8 Publication bias

Publication bias represents a significant threat to systematic reviews and meta-analyses, referring to the phenomenon whereby the nature of study results (e.g., statistical significance, effect direction) influences publication probability, thereby causing published literature to inadequately represent all completed studies. This study employed multiple methods to assess publication bias, including visual inspection of funnel plots, Egger’s regression test, S-value sensitivity analysis, and publication bias simulation analysis.

**Figure 6:**
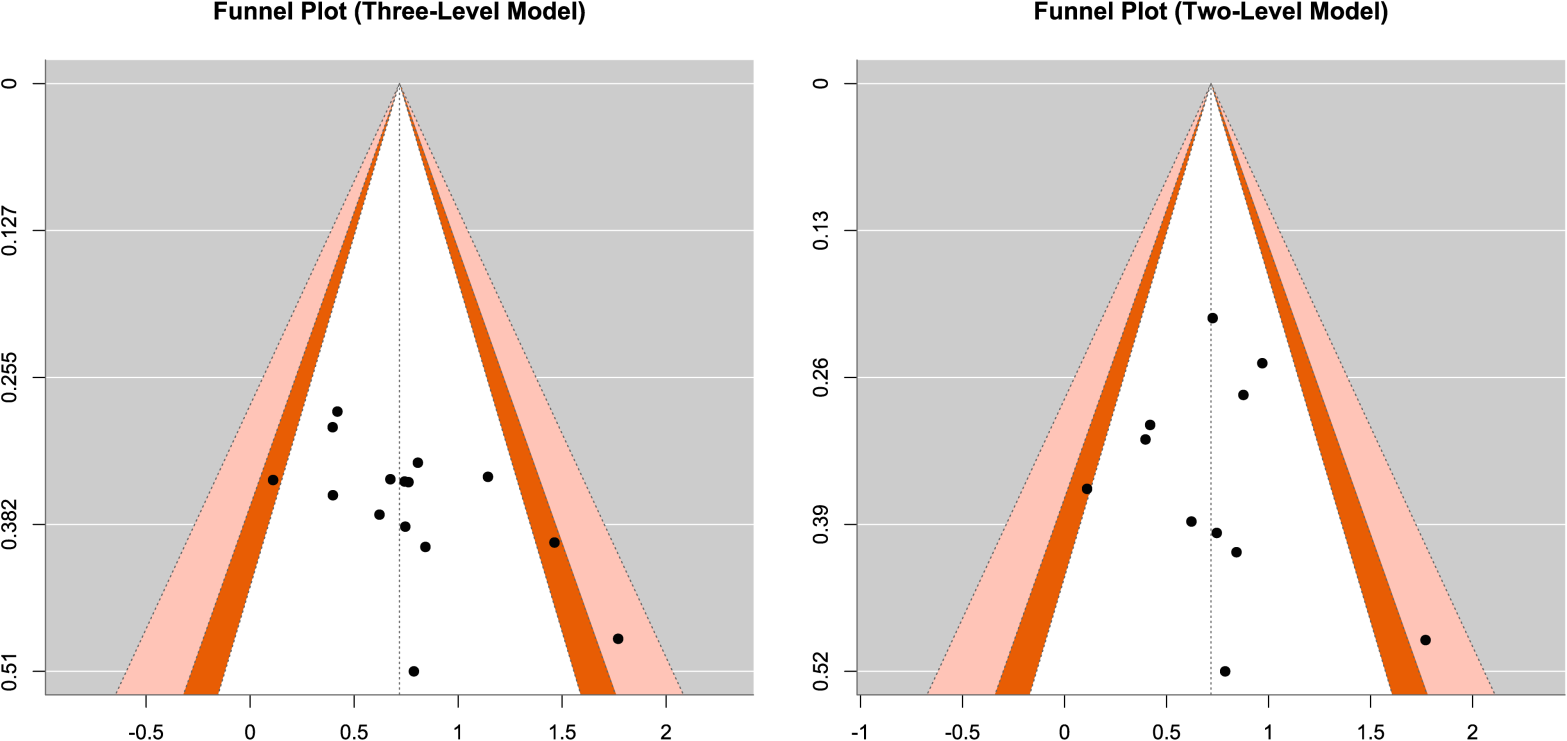
Funnel plots for publication bias in the two-level and three-level meta-analysis models.

The funnel plot (**Figure 6**) displays effect sizes on the horizontal axis and standard errors on the vertical axis, illustrating the distribution pattern of included studies. In the absence of publication bias, studies should be symmetrically distributed on both sides of the pooled effect size, forming an inverted funnel shape, with small-sample studies more dispersed and large-sample studies clustered at the top. The funnel plot for this study showed that the 15 effect sizes were essentially symmetrically distributed on both sides of the pooled effect size (SMD = 0.71), with no apparent asymmetric pattern. The contour-enhanced funnel plot further annotated effect sizes according to statistical significance levels, revealing that the distribution of significant and non-significant results did not exhibit systematic bias patterns.

Egger’s regression test assesses publication bias by testing funnel plot asymmetry. This study conducted Egger’s test using both traditional two-level random-effects models and three-level random-effects models, yielding different results. The two-level model Egger’s test showed statistical significance (t = 2.339, p = 0.019), with a regression intercept of -0.785 (95% CI [-2.053, 0.484]), suggesting possible publication bias. However, the three-level model Egger’s test was non-significant (t = 0.516, p = 0.606), with a regression intercept of 0.524 (95% CI [-0.220, 1.268]), detecting no evidence of publication bias. Considering the data structural characteristics of this study—15 effect sizes within 11 studies, with some studies reporting results from multiple measurement time points or multiple cognitive tasks—the three-level model appropriately handles the nested structure of effect sizes within studies, rendering it more reliable than the two-level model. Based on three-level model results, this study found no clear evidence of publication bias.

Synthesizing results from visual inspection of funnel plots, three-level Egger’s test, and publication bias simulation analyses, this study detected no obvious evidence of publication bias. Although the traditional two-level model Egger’s test suggested possible bias, the three-level model accounting for data nested structure did not support this conclusion. More importantly, even when conservatively assuming potential publication bias, bias-corrected effect sizes remained significant and clinically meaningful. These lines of evidence collectively support the credibility and robustness of this meta-analysis conclusion, namely that the beneficial effects of exercise interventions on inhibitory control abilities in children with ADHD are genuine rather than artifacts of publication bias.

### 3.9 Results of evidence certainty assessment

The GRADE (Grading of Recommendations Assessment, Development and Evaluation) approach was employed to systematically evaluate the quality of evidence in this meta-analysis. GRADE is an internationally recognized evidence quality assessment system that classifies evidence quality into four levels—high, moderate, low, and very low—by evaluating five downgrading factors (risk of bias, inconsistency, indirectness, imprecision, and publication bias) and three upgrading factors (large effect size, dose-response relationship, and plausible confounding factors). Since all 11 studies included in this research were RCTs, the initial evidence quality rating was high.

Among the five downgrading factors, the risk of bias domain was judged to have very serious concerns, resulting in a two-level downgrade in evidence quality. As described in Section 3.3, only 1 study (9%) among the 11 included studies demonstrated low risk of bias, a proportion far below acceptable standards; 9 studies (82%) raised some concerns, comprising the vast majority; and 2 studies (18%) had high risk of bias. The primary methodological limitations included: (1) due to the nature of exercise interventions, most studies could not implement blinding of participants and personnel, representing a systematic and difficult-to-overcome methodological deficiency that may lead to expectation effects and performance bias; (2) some studies did not implement outcome assessor blinding or had unclear blinding status, further increasing measurement bias risk; and (3) most studies lacked trial pre-registration, raising serious concerns about selective reporting. Although sensitivity analyses demonstrated stable effect sizes after excluding high-risk studies (leave-one-out analysis range: 0.668-0.760, variation of only 0.092), and no anomalous Cook’s distances or standardized residuals were detected, this statistical robustness cannot compensate for fundamental methodological deficiencies at the study design level. Considering the extremely low proportion of low-risk studies, the pervasiveness and severity of methodological problems, and the irreparable nature of absent blinding as a systematic bias source, evidence quality was downgraded two levels in this domain.

The inconsistency domain was not downgraded. Between-study heterogeneity was extremely low (I² = 0-7.3%), Cochran’s Q test was non-significant (Q = 16.31, p = 0.295), and all studies demonstrated consistent effect estimate directions, all supporting exercise interventions. Leave-one-out sensitivity analysis further confirmed high stability of effect sizes, with minimal variation across 11 iterative analyses. This indicates that included study results demonstrated high consistency, with no unexplained heterogeneity.

The indirectness domain was not downgraded. The population (6-14-year-old children and adolescents with ADHD), intervention (various forms of exercise), comparisons (usual care or no intervention), and outcomes (standardized inhibitory control tests) of included studies directly corresponded to the PICO question of this research. Although different studies employed different measurement instruments (e.g., Stroop task, Go/No-Go task, Flanker task, Simon task), these instruments are all classic paradigms for assessing inhibitory control, representing valid measurements of the same cognitive domain. The diversity of intervention types actually enhanced external validity and generalizability of results rather than constituting an indirectness issue.

The imprecision domain was not downgraded. This meta-analysis included a total sample size of 512 participants (268 in intervention groups, 244 in control groups), meeting optimal information size (OIS) requirements. The 95% CI for the pooled effect size was [0.52, 0.91], not crossing the line of no effect (SMD = 0), with the lower CI bound (0.52) still representing a medium effect, and statistical significance was very high (p < 0.001). This indicates that effect estimates possessed sufficient precision, with no imprecision issues due to inadequate sample size.

The publication bias domain was not downgraded. As described in Section 3.8, Egger’s regression test based on the three-level random-effects model detected no evidence of publication bias (t = 0.516, p = 0.606). S-value sensitivity analysis indicated that even extremely severe publication bias could not fully explain the observed effect (S-value = “Not possible”). Visual inspection of funnel plots showed relatively symmetric study distribution. Although the traditional two-level model Egger’s test showed significance (p = 0.019), considering the nested structure of the data, three-level model results were more reliable.

Regarding upgrading factors, although the pooled effect size (SMD = 0.71) constituted a medium-to-large effect, it did not reach the strict criterion for a large effect (SMD ≥ 0.8); therefore, no upgrade for large effect size was warranted. Meta-regression analyses found no evidence of dose-response relationships (see Section 3.6); therefore, no upgrade for dose-response relationship was warranted. The upgrading factor for confounding primarily applies to observational studies, whereas all studies included in this research were RCTs, with randomization controlling for known and unknown confounders; therefore, this upgrading condition was not applicable.

Synthesizing the above assessments, the evidence quality of this meta-analysis was ultimately rated as low. This means our confidence in the effect estimate is limited, and the true effect may differ substantially from the estimated effect. Low-quality evidence suggests that exercise interventions may produce improvements in inhibitory control abilities in children with ADHD, but due to pervasive methodological limitations in included studies (particularly absent blinding and lack of pre-registration), uncertainty exists regarding the magnitude of the effect size. Future high-quality studies employing more rigorous methodological designs (e.g., implementing outcome assessor blinding, conducting trial pre-registration, utilizing objective physiological indicators) may likely alter our understanding of intervention effects. Nevertheless, the directionality of current evidence is consistent, with all included studies supporting beneficial effects of exercise interventions, and effect sizes being statistically highly significant and stable. This provides preliminary but cautiously interpreted evidentiary support for exercise interventions as an adjunctive approach to improving inhibitory control abilities in children with ADHD.

## 4 Discussion

This systematic review and meta-analysis included 11 RCTs comprising 512 participants, employing a three-level random-effects model to evaluate the effects of exercise interventions on inhibitory control abilities in children with ADHD. Results demonstrated that exercise interventions produced medium-to-large improvements in inhibitory control abilities in children with ADHD (SMD = 0.71, 95% CI [0.52, 0.91], p < 0.001). This effect size approached the threshold for a large effect according to Cohen’s standards, demonstrating substantial clinical significance. Notably, between-study heterogeneity was extremely low (I² = 0-7.3%, Cochran’s Q test p = 0.295), suggesting that this effect exhibited high consistency across different study conditions. Effect estimates from all included studies consistently supported beneficial effects of exercise interventions, with no negative effects observed. Prediction interval analysis indicated that effect sizes in future studies would likely remain within the positive range, further supporting the robustness of intervention effects. Sensitivity analysis using the leave-one-out method demonstrated that the pooled effect size ranged only from 0.668 to 0.760, representing minimal relative variation, with all iterative analyses maintaining statistical significance, confirming that results were not unduly influenced by any single study. Meta-regression analyses detected no significant effects of moderating variables such as gender composition, training frequency, session duration, or total intervention duration, suggesting that exercise intervention effects demonstrate broad applicability and robustness within the parameter ranges covered by this study (1–5 sessions per week, 20–60 minutes per session, 1–24 weeks duration).

The effects of exercise interventions in improving inhibitory control abilities in children with ADHD may be realized through multiple interrelated neurobiological and psychological-behavioral mechanisms. From a neurochemical perspective, exercise-induced modulation of catecholaminergic neurotransmitter systems may represent a core mechanism for improving inhibitory control. Prefrontal cortex function depends on moderate levels of dopamine D1 receptor stimulation and norepinephrine α2A receptor stimulation, which are responsible for reducing neural “noise” and enhancing “signal,” respectively (45, 46). Pathophysiological research on ADHD patients indicates defective catecholaminergic signaling in the prefrontal cortex, closely associated with inattention and impulse control disorders (47, 48). Animal studies demonstrate that at clinically relevant doses, exercise preferentially increases norepinephrine and dopamine release in the prefrontal cortex (49). For children with ADHD, increased catecholamine excretion following exercise may improve inhibitory control function by optimizing the neurochemical environment of the prefrontal cortex and enhancing pyramidal neurons’ ability to distinguish signal from noise (50, 51). Particularly noteworthy, acute aerobic exercise can reduce reaction times and increase P3 amplitudes in children with ADHD, with these neurophysiological changes reflecting improvements in cognitive functions such as inhibitory control and working memory (52, 53).

Neurotrophic factor-mediated neuroplasticity represents another important pathway through which exercise improves cognitive function. Brain-derived neurotrophic factor (BDNF) plays a critical role in neuronal survival, synaptic plasticity, and cognitive function, with chronic exercise training increasing BDNF release and synthesis (54). BDNF is also considered to play an important role in ADHD pathophysiology, with disruption of BDNF signaling pathways associated with multiple neuropsychiatric disorders including ADHD (55). The prefrontal cortex and basal ganglia, as core neural circuits for inhibitory control, are highly sensitive to BDNF-mediated synaptic remodeling. Exercise-induced BDNF upregulation may enhance functional connectivity and information processing efficiency in these brain regions (56). Event-related potential studies found that children with ADHD who had higher physical fitness exhibited more normal P3 components (reflecting attention resource allocation and stimulus evaluation speed) during interference control tasks, suggesting positive effects of long-term exercise on neurophysiological processes (54).

From a cognitive neuroscience perspective, exercise interventions may improve inhibitory control by optimizing functional integration of the prefrontal cortex-basal ganglia circuit. Functional magnetic resonance imaging (fMRI) studies show that following exercise, adults with ADHD exhibit enhanced brain activation in parietal, temporal, and occipital regions during successful inhibition, with exercise-related increases in brain activation negatively correlated with task performance (57). This suggests that exercise may compensate for prefrontal functional deficits in ADHD patients by enhancing neural recruitment and improving neural efficiency. Regular structured exercise provides repeated stimulus-response-feedback loops, and this procedural cognitive training effect may strengthen inhibitory control-related neural networks through an “outside-in” mechanism. Elements such as rule adherence, movement sequence planning, and immediate feedback adjustment in exercise tasks constitute implicit training for executive functions. Open-skill exercises (e.g., ball sports) particularly require participants to respond in dynamically changing external environments, which closely resembles cognitive control processes required for executive function tasks (e.g., Go/No-Go, Stroop tasks).

Exercise-induced regulation of emotional and psychological states may also indirectly promote improvements in cognitive control abilities. Exercise as a physical stressor stimulates cortisol secretion, but unlike chronic psychological stress, exercise-induced cortisol elevation does not lead to long-term increases in cortisol levels. Instead, it reverses stress-induced decreases in BDNF expression, buffering against stress-related disorders such as depression (58). Bidirectional regulatory relationships exist between emotional states and executive functions, with positive emotional experiences and enhanced self-efficacy optimizing prefrontal-related cognitive processing efficiency, thereby indirectly enhancing inhibitory control abilities.

The extremely low between-study heterogeneity (I² = 0-7.3%) observed in this study warrants in-depth interpretation. The absence of heterogeneity suggests that the beneficial effects of exercise interventions on inhibitory control abilities in children with ADHD demonstrate high consistency and robustness, unaffected by significant moderation from most study and sample characteristics. However, low heterogeneity also indicates that this study may be influenced by certain systematic limiting factors that to some extent “mask” true sources of heterogeneity.

Meta-regression analyses failed to identify significant dose-response relationships, with training frequency (sessions per week), session duration, and total intervention duration all failing to significantly predict effect size variation. This result contradicts theoretical expectations based on the FITT (Frequency, Intensity, Time, Type) principle of exercise training. Possible explanations include: the intervention parameter ranges in included studies were relatively concentrated, with most studies employing moderate-intensity aerobic exercise 2–3 times per week for 30–45 minutes per session over 8–12 weeks. This parameter clustering limited the statistical power to detect dose-response relationships. Improvements in inhibitory control abilities may exhibit a “threshold effect,” whereby once exercise dosage reaches a minimum effective threshold, further dosage increases may not produce significant additional benefits. Different intervention parameters may produce similar net effects through different pathways; for example, high-frequency short-duration protocols may rely more on acute neurotransmitter modulation, while low-frequency long-duration protocols may depend more on chronic neuroplastic changes. Notably, exercise intensity, a core element of the FITT principle, lacked standardized reporting in included studies. Most studies only descriptively reported “moderate intensity” without providing objective physiological indicators (e.g., percentage of heart rate reserve or maximum oxygen uptake). This measurement heterogeneity may have masked the role of intensity as a true moderating variable.

Age and gender as potential moderating variables similarly showed no significant effects. The age range in included studies primarily concentrated on 6–14 years, precisely during the transitional stage from childhood to early adolescence. Within this developmental window, the prefrontal cortex and executive function networks undergo rapid maturation, and exercise interventions may effectively promote neuroplasticity throughout, thus no significant age effects were observed. Regarding gender, although meta-regression found no moderating effect of male-to-female ratio on effect size, male participants predominated in included studies (averaging approximately 80%). This gender imbalance limited adequate testing of gender effects. Considering that neurobiological characteristics of ADHD and exercise responses may exhibit gender differences, future studies require more balanced gender ratio designs to reveal potential gender moderating effects.

The inhibitory control measurement instruments included in this study exhibited considerable diversity, including Stroop tasks, Go/No-Go tasks, Flanker tasks, Simon tasks, and stop-signal tasks. Although all belong to the inhibitory control category, these tasks exhibit subtle differences in cognitive processing requirements and neural mechanisms. Stroop tasks primarily assess interference control, while stop-signal tasks emphasize response inhibition. The former relies more on the anterior cingulate cortex, while the latter depends more on the right inferior frontal gyrus. Most studies employed reaction time and accuracy as outcome indicators. While sensitive, these indicators may also be influenced by speed-accuracy tradeoff strategies, whereby children may improve performance by adjusting response strategies (e.g., more conservative response tendencies) rather than through genuine neurocognitive function enhancement. Future research should combine neurophysiological indicators (e.g., N2 and P3 components of event-related potentials) or neuroimaging indicators (e.g., brain activation patterns from functional magnetic resonance imaging) to more precisely characterize neural mechanism changes in inhibitory control.

Low heterogeneity may also be partially attributed to methodological homogeneity in included studies. The vast majority of studies employed similar research designs (small-sample waitlist control or usual care control), similar recruitment channels (e.g., clinical clinics or schools), and standardized diagnostic procedures (based on DSM criteria). This design homogeneity limited expression of true between-study variation. Pervasive methodological limitations in included studies (e.g., lack of participant and personnel blinding) may have introduced directionally consistent measurement bias (e.g., expectation effects or performance bias), thereby artificially reducing effect size variability. Low heterogeneity does not necessarily represent true effect consistency but may also reflect homogeneous distribution of systematic bias.

In summary, this study’s failure to identify significant sources of heterogeneity may reflect both the broad applicability and robustness of exercise intervention effects, as well as limitations from sample parameter clustering, measurement instrument heterogeneity, methodological homogeneity, and insufficient statistical power. Future research requires larger sample sizes, broader parameter coverage ranges, more refined subgroup delineation, and more standardized measurement methods to fully reveal moderating mechanisms and boundary conditions of exercise intervention effects.

The findings of this study are generally consistent with recent systematic reviews and meta-analyses on exercise intervention effects for children with ADHD, while also providing complementary evidence in certain aspects.

Wang (59) conducted a meta-analysis including 11 studies with 713 participants, demonstrating that exercise interventions significantly improved inhibitory function in children with ADHD (SMD = 0.78, 95% CI [0.45, 1.10]), an effect size highly similar to this study’s result (SMD = 0.71). Similarly, Song (60) analyzed 24 studies with 914 participants, finding that exercise interventions improved inhibitory control with an effect size of SMD = -0.50 (95% CI [-0.71, -0.29]). Although the directional notation differed, the absolute value still indicated a medium effect. Liang (61) meta-analysis (15 studies) also reported medium-to-large positive effects of exercise interventions on inhibitory control (Hedges’ g = 0.761, 95% CI [0.376, 1.146]). These consistent findings collectively support the conclusion that exercise interventions produce robust improvements in inhibitory control abilities in children with ADHD.

However, different meta-analyses exhibit discrepancies in moderating variable identification. Wang (59) subgroup analyses showed that exercise type (open-skill vs. closed-skill), frequency, intensity, duration, and intervention length all significantly moderated intervention effects, with open-skill exercises, moderate-to-high intensity, sessions exceeding 60 minutes, at least 2 sessions per week, and interventions lasting over 12 weeks demonstrating optimal effects. Mehren (62) network meta-analysis also found that open-skill activities were most effective in improving inhibitory control (SUCRA = 99.1%, SMD = 1.94), while closed-skill activities showed greater advantages in improving working memory. In contrast, this study failed to identify significant moderating variables, possibly due to multiple factors: relatively small sample size (11 studies vs. 15–24 studies in other meta-analyses), limiting statistical power to detect moderating effects; adoption of three-level meta-analysis methodology to handle nested data structures, which while improving effect size estimation accuracy, may have reduced sensitivity in identifying moderating variables; and relatively concentrated distribution of intervention parameters in included studies, lacking representation of extreme dosages.

### 4.1 Limitations

GRADE quality of evidence assessment indicated that the evidence quality of this study was low, primarily limited by pervasive methodological deficiencies in included studies. Among the 11 included studies, only 1 (9%) was rated as low risk of bias, 82% of studies raised some concerns, and 18% demonstrated high risk of bias. Due to the nature of exercise interventions, nearly all studies could not implement blinding of participants and personnel. This systematic deficiency may lead to expectation effects and performance bias, whereby expectations of participants and coaches may influence training performance and test results. Approximately half of studies did not implement outcome assessor blinding or had unclear blinding status, increasing detection bias risk, especially when using tasks requiring subjective scoring. Most studies lacked trial pre-registration, raising concerns about selective reporting. These methodological limitations may lead to systematic overestimation of effect sizes, with true effects potentially smaller than this study’s estimates. Low-quality evidence indicates that our confidence in effect estimates is limited, and future high-quality studies employing more rigorous methodological designs may alter current conclusions.

The total sample size of included studies was relatively limited (11 studies, 512 participants). While meeting OIS requirements, statistical power may be insufficient for detecting potential moderating effects (e.g., age, gender, ADHD subtype, comorbidity status). Meta-regression analyses detected no significant moderating variables, possibly partially attributable to Type II errors (false negatives) resulting from sample size limitations rather than genuine absence of moderating effects. The parameter coverage range in included studies was relatively concentrated, lacking adequate representation of extreme dosages and different exercise types (e.g., resistance training, yoga, dance), limiting comprehensive exploration of dose-response relationships and intervention type effects.

Follow-up periods in included studies were generally short, with most assessments conducted immediately post-intervention or within 4 weeks, lacking evaluation of medium-to-long-term effects (e.g., 3 months, 6 months, or longer). Whether improvements in inhibitory control abilities persist and whether they transfer to indicators with higher ecological validity such as daily functioning and academic performance remain unclear. Nearly all studies reported changes only in the single cognitive domain of inhibitory control, lacking comprehensive assessment of other executive function components (e.g., working memory, cognitive flexibility) and core ADHD symptoms (e.g., attention, hyperactivity-impulsivity). Therefore, it cannot be determined whether exercise intervention effects specifically target inhibitory control or exert broader beneficial effects on executive functions and behavioral symptoms.

From an external validity perspective, included studies primarily originated from high-income countries and regions, with participants predominantly school-aged boys and uneven distributions of ADHD subtypes and comorbidity conditions. This limits generalizability of results across different cultural backgrounds, socioeconomic conditions, age groups, and clinical characteristic populations. Intervention implementation settings, supervision intensity, and adherence monitoring methods varied considerably across included studies. These implementation factors may affect the effectiveness of exercise interventions in real-world settings.

Furthermore, due to the diversity of exercise types across studies and the limited number of studies for each specific modality, subgroup analyses by exercise type (e.g., team sports vs. aerobic vs. resistance training) were not feasible, precluding recommendations regarding the optimal exercise modality.

## 5 Conclusion

This systematic review and meta-analysis included 11 RCTs (512 participants), employing a three-level random-effects model to evaluate the effects of exercise interventions on inhibitory control abilities in children with ADHD. Results demonstrated that exercise interventions produced medium-to-large improvements in inhibitory control abilities in children with ADHD (SMD = 0.71, 95% CI [0.52, 0.91], p < 0.001), with extremely low between-study heterogeneity (I² = 0-7.3%), suggesting high consistency of effects. Sensitivity analyses, publication bias testing, and meta-regression analyses all supported the robustness of these results. However, GRADE quality of evidence assessment indicated low-quality evidence, primarily limited by pervasive methodological limitations in included studies, including absent blinding of participants and personnel, lack of outcome assessor blinding in some studies, and absence of trial pre-registration.

From a clinical practice perspective, low-quality evidence suggests that exercise interventions may represent a beneficial adjunctive approach for improving inhibitory control abilities in children with ADHD, although uncertainty exists regarding the magnitude of effect sizes. Given that exercise interventions offer advantages including high safety, strong accessibility, low cost, and potential additional health benefits (e.g., improved cardiorespiratory function, enhanced social interaction), regular structured exercise can be incorporated as one component of comprehensive ADHD management plans, provided that parents and children are fully informed of evidence limitations.

Based on the current evidence, clinicians and educators may consider incorporating structured physical exercise as an adjunctive intervention for children with ADHD. While the optimal exercise prescription cannot be precisely determined from the available data, the included studies suggest that moderate-intensity aerobic exercise performed 2–3 times per week, with sessions lasting 30–60 minutes, over a period of 8–12 weeks, may be a reasonable starting point. Given the heterogeneity in exercise types across studies (including aerobic exercise, exergaming, ball sports, and swimming), no specific exercise modality can be preferentially recommended at this time. Future research with standardized protocols is needed to establish more precise clinical guidelines.

## Data Availability

The original contributions presented in the study are included in the article/**Supplementary Material**. Further inquiries can be directed to the corresponding author.
